# Correlation networks visualization

**DOI:** 10.3389/fpls.2012.00240

**Published:** 2012-10-29

**Authors:** Nicholas Provart

**Affiliations:** Department of Cell and Systems Biology/Centre for the Analysis of Genome Evolution and function, University of TorontoToronto, ON, Canada

**Keywords:** coexpression analysis, network visualization, hypothesis generation, transcriptomics, protein–protein interactions

## Abstract

New, *in silico* ways of generating hypotheses based on large data sets have emerged in the past decade. These data sets have been used to investigate different aspects of plant biology, especially at the level of transcriptome, from tissue-specific expression patterns to patterns in as little as a few cells. Such publicly available data are a boon to researchers for hypothesis generation by providing a guide for experimental work such as phenotyping or genetic analysis. More advanced computational methods can leverage these data via gene coexpression analysis, the results of which can be visualized and refined using network analysis. Other kinds of networks of, e.g., protein–protein interactions, can also be used to inform biology. These networks can be visualized and analyzed with additional information on gene expression levels, subcellular localization, etc., or with other emerging kinds information. Finally, cross-level correlation is an area that will become increasingly important. Visualizing these cross-level correlations will require new data visualization tools.

## INTRODUCTION

The past decade of plant research has seen an unprecedented increase in the amount of data being generated from various levels of an organism. Advances in genome sequencing, gene expression profiling, determining all-by-all interactomes, and other areas, have allowed new, *in silico *ways of generating hypotheses, in addition to traditional and powerful forward genetic screens and techniques. These methods have been used to investigate different aspects of plant biology, especially at the level of transcriptome, from tissue-specific expression patterns at the centimeter scale to those in as little as a few cells. While such publicly available data can be used on their own by researchers to generate hypothesis, one of the most powerful methods for hypothesis generation to date is gene coexpression analysis. Here, the correlation in expression pattern between pairs of genes is measured, and those exhibiting strong correlations are “joined” in a graphical representation to create a network, which can be visualized with graph network viewers. Other kinds of networks of, e.g., protein–protein interactions, a kind of biological, *in vivo *correlation, can also be used to inform biology. These networks can be visualized and analyzed with additional information on gene expression levels, subcellular localization, etc., or with other kinds of information just becoming available, such as patterns of positive selection along a gene, genes with similar patterns of expression in equivalent tissues in other species, etc. Finally, cross-level correlation is an area that will become more important as genome wide association studies (GWAS) could be used to link genotype to environmental factors or perturbations through changes in the transcriptome, epigenome, or other ‘omes of a plant. Visualizing these cross-level correlations will require new data visualization tools, the most promising of which will allow the plant at all levels to be an “app” on a smart phone, laptop computer, or scientific conference video wall. Such efforts will serve to make the proposed open access/open source Arabidopsis Information Portal, in which zoomable user interfaces and powerful data analysis and visualization algorithms will be an important part, an immediate resource not only for *Arabidopsis* researchers, but for all plant biologists worldwide.

## GENE EXPRESSION VISUALIZATION

Large amount of gene expression data for *Arabidopsis thaliana *and other plants have been generated in the past decade, and these can be examined to provide further understanding about one’s gene(s) or biological system of interest. One can use such data to ask questions such as: within my gene’s family, are family members uniquely expressed in specific organs/tissues or is one uniquely induced in expression by a specific stress? Such data can be explored with several easy-to-use web-based tools, such as Expression Browser (Toufighi et al., 2005) or electronic Fluorescent Pictograph (eFP) Browser (Winter et al., 2007) at the Bio-Array Resource (BAR), Genevestigator (Zimmermann et al., 2004), the TileViz tool developed for the At-TAX data set generated by Laubinger et al. (2008), and others. The use of these powerful tools is described in a review by Brady and Provart (2009). While such gene expression visualization tools can be very useful for some biological questions, in order to identify patterns of correlation between different genes using these tools one would have to manually examine outputs looking for genes with similar patterns of expression, a rather onerous and subjective task. Thus it is advantage to use computational methods to identify such coexpressed genes. Coexpressed genes can be used as a “primary screen” to identify novel genes involved in one’s biological process of interest, and examples of such new insights are described in Usadel et al. (2009).

## GENE COEXPRESSION NETWORKS

**FIGURE 1:**
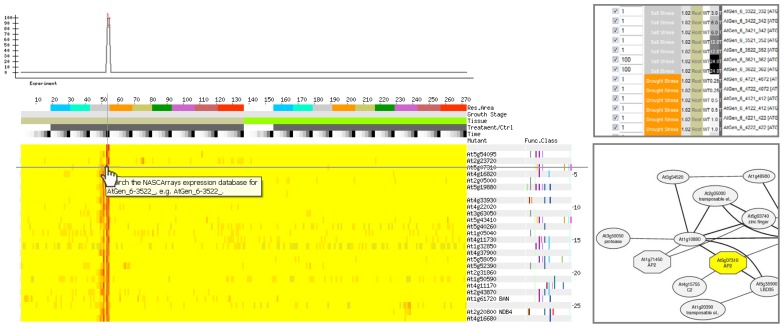
**A gene may be identified having an expression pattern as shown in the top left.** Alternately, a vector can be designed that exhibits a desired behavior. Coexpression analysis on an abiotic stress compendium produced by Kilian et al. (2007) returns genes exhibiting similar patterns of expression. In this example, only genes exhibiting strong expression in roots after 24 h of exposure to salt as specified using the *AtGenExpress Stress Set *and Expression Angler (Toufighi et al., 2005) running in the *Subselect and Custom Bait *mode (top right), are returned by the analysis, as shown in the heatmap on the bottom left. The partial network on the bottom right is an alternate depiction of the relationship between one of the output genes from this example, At5g07310 – whose gene product possesses an AP2 domain, and other genes in a condition-independent coexpression analysis provided by ATTEDII (Obayashi et al., 2009). In this network representation, nodes represent genes, and the edges represent a significant interaction as defined by coexpression scores: the thicker the edges the better the coexpression score.

Four components are necessary for conducting a gene coexpression analysis. These are: (1) a collection of gene expression profiles across as many genes as possible from different samples and/or different perturbations; (2) a method for computing expression pattern similarity; (3) a way for assessing the degree of significance of expression pattern similarity; and (4) a tool to visualize and analyze statistically significant coexpression patterns. A recent review by Usadel et al. (2009) describes how the selection of gene expression profiles to create a collection (or “compendium”) of profiles for coexpression analysis can influence the results that one gets from such an analysis. It is possible to select samples in a “condition-independent” or “condition-dependent” manner. In some cases, such as SeedNet (Bassel et al., 2011), it clearly makes sense to use data sets from specific samples (seeds in that case), while in other, more exploratory cases, condition-independent data sets can be used as a starting point for hypothesis generation. The Usadel et al. (2009) article also describes commonly used metrics for assessing expression pattern similarity. The most commonly used metrics are the Pearson’s correlation coefficient (PCC), along with Spearman’s (Rank) correlation coefficient, and mutual information. Further, the Usadel et al. (2009) review also describes ways of assessing significance of coexpression scores. It should be pointed out that if large numbers of data sets are used when computing expression pattern similarity, PCC values of as low as 0.2 will have good *P *values. Thus it is often useful to use a higher cutoff, and to consider the *r*^2^ value, achieved by squaring the PCC score, for the purpose of selecting coexpressed genes. The *r*^2^ value reflects the amount of variance in common. Thus a pair of genes with a coexpression score of 0.2 as measured by the PCC will have an *r*^2^ value of (0.2)^2^ = 0.04, or only 4% of variance in common. This fact also often explains why a PCC value of 0.75 is commonly used as a cutoff, because then the *r*^2^ value would be 0.56, meaning that genes exhibiting such a score would have 56% of their variance in common. This seems biologically “meaningful” in the sense that this variance might be directed by *cis*-regulatory elements in common in the promoters of coexpressed genes. In terms of visualizing expression patterns, often it is instructive to examine heatmap outputs for all of the highly correlated genes for a given query gene, or for those matching a desired gene expression pattern, as shown in **Figure 1**. When the values for coexpression are significant between several of the genes in the single gene query example just mentioned, it is also useful to depict these as a graph network as shown in **Figure 1**.

The network depicted in **Figure 1** can be generated using the ATTEDII coexpression tool (Obayashi et al., 2009). Nodes that are not related to the original query gene may be added to the network based on precomputed coexpression data in the ATTEDII database. Another nice feature of the ATTEDII database is the ability to easily explore coexpression scores in a condition-independent compendium or in several focused condition-dependent compendia, such as stress, hormone treatments, etc.

Other useful tools for generating and visualizing gene coexpression networks in *Arabidopsis* are CressExpress (Srinivasasainagendra et al., 2008), PlaNet (Mutwil et al., 2011), and CSB.DB (Steinhauser et al., 2004). With each of these it is possible to easily retrieve data for use in generating a coexpression network, which may be visualized online or with network viewers as described in the next paragraph. A couple of other of tools, which are not strictly coexpression-based, are also useful for generating correlation networks. GeneMANIA (Mostafavi et al., 2008) and AraNet (Lee et al., 2010) permit coexpression data to be used to connect and extend genes in a network. Further information can be used to deduce new members of a network. Such information includes subcellular localization, protein–protein interaction data, and shared protein domains. Another tool was recently developed for exploring “gene-sharing networks” for tissues that are connected based on the degree of shared, leptokurtically expressed genes, i.e., genes that are preferentially expressed in certain tissues (Li et al., 2012).

Once a coexpression network has been generated, for example, using the ATTEDII database or by a custom “all-by-all” analysis, as was the case for SeedNet, it is often useful to import the network into a powerful network analysis tool, Cytoscape (Shannon et al., 2003; Kohl et al., 2011). This is easily done from an Excel table containing the interactors (i.e., genes exhibiting a strong correlation in their expression patterns) in two columns along with their interaction strength (e.g., coexpression score). Using tools within Cytoscape, it is possible to analyze the network to identify nodes (i.e., genes) that are “hubs”, meaning these genes have large numbers of coexpression partners. These hub nodes may be analyzed in conjunction with other data, such as whether the nodes genes exhibit increased or decreased expression levels under particular conditions, to identify significant hub genes (Horvath and Dong, 2008). These significant hub genes may play important roles biologically. In the case of SeedNet, 50% of the genes identified as hub genes were accurately predicted to be regulators of germination based on follow up biological experiments, in contrast to a 22% accuracy rate based on candidates identified by differential gene expression analysis alone. “Modules”, that is, genes coexpressed as a group, which would have many coexpression connections between all of the genes in the module, can be identified using the MCODE algorithm (Bader and Hogue, 2003). Further analysis of the genes in a module can be done using several Gene Ontology (Ashburner et al., 2000) term enrichment analysis plugins available for Cytoscape, to see whether the genes in a given module are involved with a particular biological process or molecular function. Other graph network visualization tools such as Pajek (Batagelj and Mrvar, 1998) or Biolayout (Theocharidis et al., 2009) can also be used to visualize networks. The aforementioned databases and visualization tools are summarized with their availability and features in **Table 1**.

**TABLE 1:** Selected correlation network databases and tools.

URL and comments	
Correlation network DB	
ATTEDII (Obayashi et al., 2009)	http://atted.jp/; explore condition-independent coexpression networks for up to 100 genes in *Arabidopsis* using the NetworkDrawer tool. Coexpression analyses may also be performed for rice.
CressExpress (Srinivasasainagendra et al., 2008)	http://cressexpress.org; generate condition-independent coexpression analyses or custom condition-dependent coexpression analyses for *Arabidopsis* with up to 30 genes. Results are easily imported into Cytoscape for visualization.
PlaNet (Mutwil et al., 2011)	http://aranet.mpimp-golm.mpg.de/; use this tool to explore condition-independent coexpression networks in seven plant species. Networks are displayed as static SVG images, but networks may also be downloaded for easy viewing and further manipulation in Cytoscape or Pajek.
CSB.DB (Steinhauser et al., 2004)	http://csbdb.mpimp-golm.mpg.de/; use this tool to explore both condition-independent or condition-dependent coexpression networks in *Arabidopsis* for up to 60 genes. Networks may be immediately viewed as images, or downloaded for further manipulation into the visualization tools below.
GeneMANIA (Mostafavi et al., 2008)	http://genemania.org/; this tool allows functional network generation in *Arabidopsis* based on user-selected or default expression data sets, protein–protein interactions, subcellular localization, shared protein domains, etc. Results are easily visualized via an embedded Cytoscape Web (Lopes et al., 2010) application.
SeedNet (Bassel et al., 2011)	http://bree.cs.nott.ac.uk/arabidopsis/; explore condition specific (i.e., seed-expressed) gene networks from *Arabidopsis* in a custom network explorer.
AraNet (Lee et al., 2010)	http://www.functionalnet.org/aranet/; like GeneMANIA this tool allows functional network generation in *Arabidopsis*. Results may be visualized via activation of Cytoscape Web.
**Network visualization tools**	
Cytoscape (Shannon et al., 2003; Kohl et al., 2011)	http://www.cytoscape.org/; use this powerful open-source desktop tool to visualize coexpression and other networks, such as those generated by protein–protein interaction studies. Nodes and edges may be appended with additional, user-defined information.
Biolayout (Theocharidis et al., 2009)	http://biolayout.org/; the current iteration of this desktop tool, Biolayout *Express*^3^^D^, permits visualization of coexpression and other networks in three-dimensional space. Cytoscape “.sif” files may be imported into the tool.
Pajek (Batagelj and Mrvar, 1998)	http://pajek.imfm.si/doku.php

## TRANSCRIPTOME DATA IN OTHER KINDS OF CORRELATION NETWORKS

Elucidating protein–protein interaction networks has proven to be useful for the identification of novel members of a biological process. In the case of *Arabidopsis thaliana*, many groups have used biochemical or yeast-based methods, such as the yeast two hybrid system (Fields and Song, 1989), to determine whether or not small subsets of proteins interact with one another. The largest application of the yeast two hybrid method to date for plants was published last year by the Arabidopsis Interactome Mapping Consortium (2011), and covers an estimated 2% of the potential protein–protein interactions in this species. Computational methods can also be used to predict protein–protein interactions in a species based on the presence of interacting orthologs in other species (Geisler-Lee et al., 2007). An important caveat to protein–protein interaction analysis methods, especially those based on protein expression in heterologous systems such as yeast, is that even though proteins can interact *in vitro *or *in vivo*, doesn’t mean that they necessarily interact *in vivo *in every tissue of the plant or under every perturbation. Other factors, such as expression levels, protein degradation, and subcellular localization, are important for an interaction to occur. For instance, if two proteins can physically interact based on yeast two hybrid data, but one of these proteins is not expressed in a given tissue or organ *in planta*, then that interaction cannot occur in that tissue or organ. Likewise, proteins that are not localized in the same subcellular compartment would seem to have a lower likelihood of being able to interact *in planta*, although it is clear that proteins, especially signaling proteins, can move between compartments.

**FIGURE 2:**
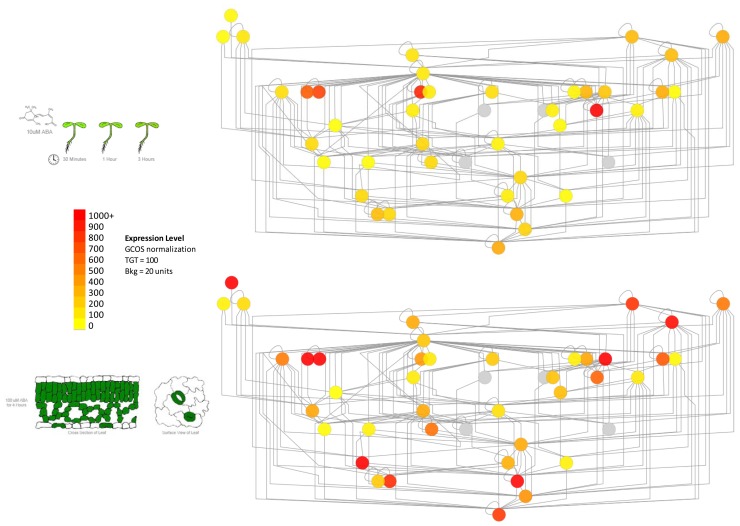
**Using gene expression data to delineate subnetworks within larger protein–protein interaction networks determined by the yeast two hybrid method.** Left panel: part of the vesicle trafficking PPI interactome, with nodes colored according to average expression level of the corresponding genes in seedling tissue (root and shoot) treated with 10 μM ABA (Goda et al., 2008). Right panel: the same network colored according to the average expression level of the same genes in purified mesophyll and guard cells treated with 100 μM ABA for 4 h (Yang et al., 2008). The genes for different proteins exhibit stronger expression levels in these distinct tissues, highlighting potential protein–protein interaction subnetworks in these different kinds of tissues. Gray nodes denote that no expression information is available.

In order to address the above caveat, it is possible to overlay subcellular localization data or gene expression data onto protein–protein interaction networks. Such data are available in the former case from SUBA, the *Arabidopsis* subcellular database (Heazlewood et al., 2006), and in the latter from extensive expression repositories at NCBI’s GEO (Edgar et al., 2002), the BAR (Toufighi et al., 2005), Genevestigator (Zimmermann et al., 2004) and other sources. An example of how such expression data can be used to delineate subnetworks within PPI networks can be seen in **Figure 2**. The network shows some proteins involved in vesicle trafficking, based both on literature-documented and predicted protein–protein interactions (Geisler-Lee et al., 2007).

As shown in **Figure 2**, only certain members of the “supernetwork” of all possible protein–protein interactions are expressed in either seedlings or guard cells. While further experiments are necessary to show whether the nodes with higher expression levels, shown in red or orange in the figure, are actually key to vesicle trafficking in these tissues, such information can clearly be useful in terms of ordering T-DNA knockout lines of specific genes (Alonso et al., 2003) and identifying under which conditions or in which tissues to look for a phenotype.

## CORRELATION NETWORKS ACROSS DIFFERENT SPECIES

The above two sections have highlighted two ways in which correlation networks can be used to guide biology in one very well studied species, *Arabidopsis thaliana*. Recent work by Mutwil et al. (2011) has computed coexpression networks in different plant species, and has used similar coexpressed network vicinities to help identify functional homologs across species. These data are accessible through the PlaNet tool. CoP by Ogata et al. (2010) and STARNET2 by Jupiter et al. (2009) offer a similar functionality. In a slightly different approach, Patel et al. (2012) have used gene expression atlases for seven plant species – *Arabidopsis*, soybean, *Medicago truncatula*, poplar, barley, maize, and rice – to identify equivalent tissues between these species. Based on these tissue equivalencies, the authors then computed the expression pattern similarity scores for a set of homologs from one species to a homolog from another species. The homolog exhibiting the best expression pattern similarity score is termed the “expressolog”. The authors showed that the number of instances in which the expressologs are not the best sequence similarity matches ranges from a low of 15.4% between poplar and *M. truncatula*, to a high of 50.7% between barley and soybean. The expression pattern and sequence similarity scores may be viewed with the Expressolog Tree Viewer tool available at http://bar.utoronto.ca. Given the complexity of plant genomes in terms of whole genome and segmental duplication events (Arabidopsis Genome Initiative, 2000; Jiao et al., 2011), it is perhaps not surprising that expression patterns and sequences can follow different evolutionary trajectories. Interestingly, Movahedi et al. (2011) postulate that coexpression networks have undergone concerted rewiring in *Arabidopsis* as compared to rice.

## CORRELATION NETWORKS ACROSS DIFFERENT SCALES AND A LOOK TO THE FUTURE

It is becoming easier to produce transcriptome data for specific cell types, following the protocol initially developed in Philip Benfey’s lab (Birnbaum et al., 2003). A high resolution spatio-temporal map of root development covering more than 120 different cell types and maturity stages provides unprecedented insight into root biology (Brady et al., 2007). Recent efforts to generate epigenomic information using bisulfite sequencing or “BS-seq”, first established by Lister et al. (2008) can be extended down to the cell and even allele level (Peng and Ecker, 2012). Further information on the genomes of ecotypes of *Arabidopsis* is being generated by the 1001 Arabidopsis Genomes effort (Weigel and Mott, 2009). Correlating genomic and epigenomic variation with gene expression levels will provide new insight into plant biology. For instance, it was recently shown by Dowen et al. (2012) that plants differentially methylate their genomes in response to biotic stress, and that these patterns are closely associated with differential gene expression responses. Incorporating transcriptome data into GWAS efforts will also allow greater insight. Such an approach was recently published by Gan et al. (2011). The challenge for researchers will be to easily tap into such data sets to ask whether their gene or system of interest might be under some kind of regulation described in the literature, and to visualized these data sets integratively. It is hoped that the development of a new Arabidopsis Information Portal (International Arabidopsis Informatics Consortium, 2012) will easily enable such analyses, and that the further development of visualization tools, such as a “multi-omics” plugin for Cytoscape (Enjalbert et al., 2011), will allow us to visualize the results in a comprehensible manner.

## Conflict of Interest Statement

The author declares that the research was conducted in the absence of any commercial or financial relationships that could be construed as a potential conflict of interest.
